# Ibrutinib versus bendamustine plus rituximab for first-line treatment of 65 or older patients with untreated chronic lymphocytic leukemia without del(17p)/TP53 mutation in China: a lifetime economic research study

**DOI:** 10.1186/s12913-023-10402-0

**Published:** 2023-12-05

**Authors:** Yuan Hong, Xichuang Chen, Yuanquan Hong, Xingfang Xiao, Yan Wang, Xiaohong You, Jingyi Mi, Tao Zhou, Panpan Zheng, Zhihu Huang

**Affiliations:** 1 Department of Pharmacy, Affiliated Children’s Hospital of Jiangnan University (Wuxi Children’s Hospital), Wuxi, Jiangsu China; 2 Department of Pharmacy, Wuxi Ninth People’s Hospital Affiliated to Soochow University (Wuxi Orthopedic Hospital), Liangxi Road 999, Wuxi, Jiangsu 214062 China; 3 Department of Sports Medicine, Wuxi Ninth People’s Hospital Affiliated to Soochow University (Wuxi Orthopedic Hospital), Wuxi, Jiangsu China; 4 Department of Pharmacy, Ningbo No. 6 Hospital, Ningbo, Zhejiang China; 5 Department of Oncology, Wuxi Ninth People’s Hospital Affiliated to Soochow University (Wuxi Orthopedic Hospital), Wuxi, Jiangsu China

**Keywords:** Chronic lymphocytic leukemia, Cost effectiveness, Ibrutinib, Bendamustine, Rituximab, Quality adjusted life-year

## Abstract

**Background:**

The incidence and mortality rates of patients with chronic lymphocytic leukemia (CLL) in China have recently increased. This study performed a long-term economic evaluation of the first-line treatment strategies ibrutinib (IB) or bendamustine (BE) plus rituximab (RI) for previously untreated older patients with CLL without the del(17p)/TP53 mutation in China.

**Methods:**

Based on clinical data from large, randomized trials, a Markov model including four disease states (event-free survival, treatment failure, post-treatment failure, and death) was used to estimate the incremental costs per quality adjusted-life year (QALY) gained from the first-line IB strategy versus the BE plus RI strategy over a 10-year period. All costs were adjusted to 2022 values based on the Chinese Consumer Price Index, and all costs and health outcomes were discounted at an annual rate of 5%. Sensitivity analysis was performed to confirm the robustness of base-case results.

**Results:**

Compared to the first-line BE plus RI strategy, first-line IB treatment achieved 1.17 additional QALYs, but was accompanied by $88,046.78 (estimated in 2022 US dollars) in decremental costs per patient over 10 years. Thus, first-line treatment with IB appeared to have absolute dominance compared to the BE plus RI strategy. Sensitivity analysis confirmed the robustness of these results.

**Conclusions:**

The first-line treatment with IB is absolutely cost-effective compared to the first-line BE plus RI treatment strategy for 65 or older patients with CLL without the del (17p)/TP53 mutation from the Chinese payer perspective. Therefore, it is strongly recommended that Chinese health authorities select the former strategy for these CLL patients.

## Background

Chronic lymphocytic leukemia (CLL) is the most common form of adult leukemia in Western countries with an age-adjusted incidence rate (AAIR) of approximately 4.5 per 100,000 individuals [1], although the AAIR is 5- to 10-times lower in East Asians, Asian Indians, and Amerindians [2–5]. However, a burden study recently demonstrated that East Asians had the highest growth or mortality rate among CLL patients, with an estimated increase in annual percentage change of 7.98 or 4.34 times in 1990 and 2019, respectively [6]. Furthermore, of 204 countries and territories, China was one of the top 3 countries with the highest incidence or mortality rate of CLL in 2019 [6], and thus, optimizing healthcare intervention from socio-economic perspective may be of highest priorities to realize better treatment strategies and cares for patients with CLL in China.

CLL is most frequently diagnosed in patients aged 65–74 years of age and the median age at diagnosis is 69 years, with only 2.0% of patients diagnosed under the age of 45 [1], remaining significant unmet medical needs. The latest Chinese CLL guidelines recommend using Bruton tyrosine kinase inhibitors (BTKi), such as ibrutinib (IB), zanubrutinib (ZB), or chemoimmunotherapy with bendamustine (BE) plus rituximab (RI) for previously untreated Chinese patients with CLL aged 65 years or older without del(17p)/TP53 mutation [7, 8]. However, chemoimmunotherapy is generally associated with toxic effects, and the risk of toxicity increases with age. Furthermore, unlike chemoimmunotherapy regimens (usually lasting 6 months), BTKis are oral drugs that are more convenient for prolonged treatment, despite the additional treatment costs of patients with CLL [9, 10]. As reported, the IB regimen is not considered a cost-effective approach for the first-line treatment of untreated CLL patients in the United States and the United Kingdom compared to chemoimmunotherapy, mainly due to its high monthly costs [11–13]. However, IB was introduced to the Chinese National Medical Insurance Negotiation Directory in 2018 with significantly reduced costs. China remains a developing country with a population of nearly 1.4 billion. The gross domestic product (GDP) on mainland China in 2022 was only approximately $12,741 [14]; thus, it is worth exploring whether IB as a first-line strategy for the treatment of Chinese patients with CLL is cost-effective. However, there has been no localized health economic study specific to the Chinese population.

Therefore, the objective of this study was to evaluate the cost-effectiveness of first-line treatment with IB compared with BE plus RI for previously untreated Chinese patients with CLL aged 65 years or older without del(17p)/TP53 mutation using local data. This finding will provide a theoretical rationale and evidence to support treatment policies by Chinese decision makers.

## Methods

The most recent Chinese Guideline of the Pharmacoeconomic Evaluations and Manual (2020) was used to perform our cost-effectiveness analysis [15].

### Study population and settings

For the economic evaluation, untreated Chinese patients with CLL aged 65 years or older not harboring the del(17p)/TP53 mutation were enrolled. These patients reflected an individual cohort in the ALLIANCE (A041202) phase 3 randomized controlled trial that compared first-line IB therapy with BE plus RI in these patients [16]. The characteristics of this cohort were as follows: a median age of 70–71 years, 66.3% were male, 54.2% were classified as high-risk, 60.4% had an unmutated immunoglobulin variable heavy chain (IGVH) gene, and 6.4% of all patients had a 17p deletion [16].

This cohort of patients would require 1 of the 2 regimens under comparison to manage CLL (first-line treatment): IB or BE plus RI. Both initial strategies were derived from standard treatments in the ALLIANCE (A041202) trial [16]. Patients in the IB group received IB at 420 mg per day orally until they experienced disease progression or unacceptable toxic effects. The patients in the BE plus RI group received BE intravenously at 90 mg/m^2^ body surface area (BSA) per day on days 1 and 2 of each cycle for 6 cycles plus RI intravenously at 375 mg/m^2^ BSA per day on the day before day 1 of cycle 1, and then 500 mg/m^2^ of BSA daily on day 1 of cycles 2 through 6. Since a low oral low dose of lenalidomide (LE) was generally considered a maintenance strategy after chemoimmunotherapy [7, 8, 17], we used an increased dose of LE, administering 5, 10, or 15 mg per day on days 1 through 28 in cycle 7, cycles 8 to 12, or each cycle thereafter, respectively [18]. Patients who experienced disease progression after first-line treatment received subsequent therapies. According to the latest Chinese CLL guidelines [7, 8], we selected ZB [19] and orelabrutinib (OB) [20] or IB [21, 22] and ZB [19] as second and third line treatment schemes for the IB or BE plus RI groups, respectively. Progressed patients received ZB, OB, or IB at 320 mg, 150 mg, or 420 mg per day orally, respectively, according to respective clinical trials [19–21]. If the disease progressed, patients in both groups continued treatment with allogeneic hematopoietic stem cell transplantation (allo-HSCT) as the last strategy [19–21]. Additionally, we assumed that patients could enter the best supportive care (BSC) health state after disease progression following any line treatment regimen: transition probabilities of first- to third-line treatment regimens derived from a previous study [23], yet entered completely after disease progression in allo-HSCT.

### Model structure

**Fig. 1:**
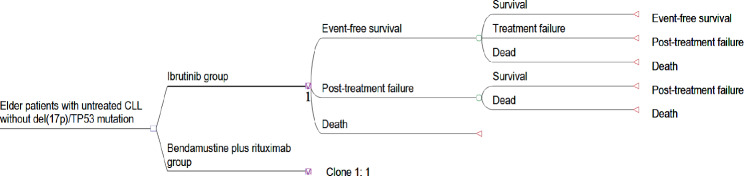
The Markov model of older patients untreated chronic lymphocytic leukemia without the del(17p)/TP53 mutation. The model structure included four mutually exclusive health states: event-free survival (EFS), treatment failure (TF), post-treatment failure (PF), and death. The duration of each Markov cycle was 1 month in the first 4 years and then 1 year thereafter. The EFS state was the entrance and the TF state was transient, so patients could automatically move to the PF state of the cycle after relapse or TF

We developed a Markov state transition model with TreeAge Pro 2011 (TreeAge Software, Williamstown, MA), embracing the 4 mutually exclusive health states shown in Fig. 1: event-free survival (EFS), treatment failure (TF), post-treatment failure (PF), and death [24]. The duration of each Markov cycle was 1 month in the first 4 years and then 1 year thereafter. The EFS state was the entrance and the TF state was transient, so patients would automatically move to the PF state of the cycle after relapse or TF. Furthermore, we assumed that treatment-induced severe adverse events (SAEs) of grade 3 and above occurred during the first month of treatment in the EFS state. This economic research study was constructed based on a literature review and modeling techniques; thus, written consent was unnecessary.

### Perspective, time horizon, and discounting

This evaluation was performed from the perspective of the Chinese health care providers. As the Chinese average life expectancy was 77.93 years according to the National Bureau of Statistics in 2020, we used a lifetime horizon of 10 years in this model, considering the median age of the cohort was 70–71 years as derived from a pivotal study [16]. Costs and health outcomes were discounted at 5% annually [15], and converted into a monthly discount for the running of the first 48-month Markov cycles [9].

### Transition probabilities

**Fig. 2:**
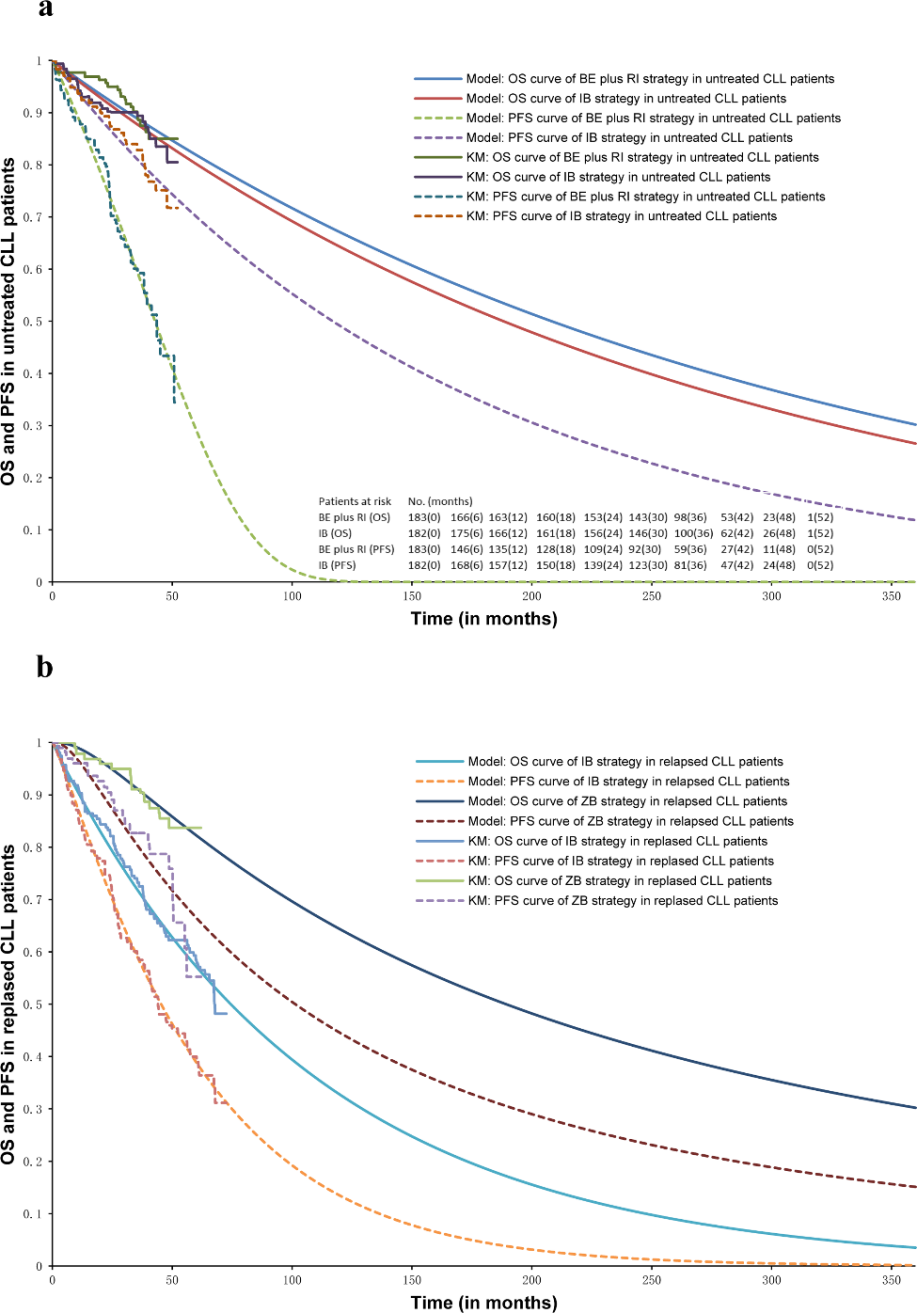
Survival outcomes. Best parametric survival distributions of overall survival (OS) and progression-free survival (PFS) probabilities in patients with untreated (a) or relapsed (b) chronic lymphocytic leukemia (CLL). The embedded table in Fig. 2a showed number of patients at risk over time sourced from the ALLIANCE (A041202) trial [16]. BE: bendamustine; RI: rituximab; IB: ibrutinib; ZB: zanubrutinib

Transition probabilities determined the way in which transitions between different health states were achieved in patients with CLL in the Markov model. The Kaplan–Meier (KM) general survival curves (OS) and progression-free survival (PFS) of the treatment strategies of IB [16] and BE plus RI [16] in patients with untreated CLL, or IB [22] and ZB [19] in patients with relapsed CLL from pivotal studies were digitized using Engauge Digitizer software 6.1 [25]. An approximation of individual patient data (IPD) was reconstructed based on the algorithm of Guyot et al. [26] using the digitized KM survival curves and information on the number of patients at risk of events at several follow-up times. IPD was used to parameterize the OS and PFS curves to infer the extrapolation values of the survival curves beyond the follow-up period reported in clinical trials. Standard parametric models (exponential, Weibull, Gompertz, Gamma, lognormal, and loglogistic) were fitted to the data extracted from the KM curves using R for Statistical Computing (R-Foundation, Peking University, China), and we selected the best parameter distribution using Akaike’s information criterion, Bayesian information criterion, and visual judgment for inclusion in the Markov model. The estimated parameters are presented in Table 1, and the best parametric survival distributions are illustrated in Fig. 2. Furthermore, we used the value of the objective response rate (ORR) of OB treatment in relapsed or refractory patients with CLL in this model, as survival curves were not available at present [20]. As the number of allo-HSCT patients at risk in cumulative incidence curves of relapse/progression and non-relapse mortality was lacking, we used Engauge Digitizer software 6.1 to digitize data in a 10-year period and converted them to transition probabilities performed by the model [27].

**Table 1:** Model clinical parameters

Variables	Estimate	Lower Bound^a^	Upper Bound^a^	Distribution	Reference
Transition probabilities^b^					
PFS for first-line IB strategy in untreated CLL patients	Exponential: rate = 0.00592451	-	-	Fixed in PSA	Woyach et al., 2018 [16]
PFS for first-line BE plus RI strategy in untreated CLL patients	Gompertz: shape = 0.02325928, rate = 0.00943535	-	-	Fixed in PSA	Woyach et al., 2018 [16]
PFS for second-line IB strategy in replased CLL patients	Gamma: shape = 1.1802239, rate = 0.0191373	-	-	Fixed in PSA	Byrd et al., 2014 [21], Munir 2019 [22]
PFS for second or third-line ZB strategy in replased CLL patients	Gompertz: shape = 0.0341335, rate = 0.00268049	-	-	Fixed in PSA	Cull et al., 2022 [19]
OS for first-line IB strategy in untreated CLL patients	Exponential: rate = 0.00368296	-	-	Fixed in PSA	Woyach et al., 2018 [16]
OS for first-line BE plus RI strategy in untreated CLL patients	Exponential: rate = 0.00332761	-	-	Fixed in PSA	Woyach et al., 2018 [16]
OS for second-line IB strategy in replased CLL patients	Exponential: rate = 0.00100394	-	-	Fixed in PSA	Byrd et al., 2014 [21], Munir 2019 [22]
OS for second or third-line ZB strategy in replased CLL patients	Lognormal: log of mean = 1.59, log of SD = 164.93	-	-	Fixed in PSA	Cull et al., 2022 [19]
ORR of OB strategy in patients with relapsed or refractory CLL	0.91	0.83	0.96	Beta	Xu et al., 2020 [20]
Probability of receiving BSC after progressing from first-line treatment	0.15	0.11	0.18	Beta	Else et al., 2016 [23]
Probability of receiving BSC after progressing from second or third-line treatment	0.19	0.14	0.24	Beta	Else et al., 2016 [23]
Treatment cost, $					
Medications, per milligram^c^					
IB	0.20	0.15	0.25	Gamma	Local charge
ZB	0.33	0.25	0.41	Gamma	Local charge
OB	0.71	0.53	0.89	Gamma	Local charge
BE	8.57	6.43	10.72	Gamma	Local charge
RI	3.59	2.70	4.49	Gamma	Local charge
LE	12.07	9.05	15.09	Gamma	Local charge
Medications, per month^c^					
IB	2360.36	1770.27	2950.45	Gamma	Local charge
ZB	2940.04	2205.03	3675.05	Gamma	Local charge
OB	2995.66	2246.74	3744.57	Gamma	Local charge
BE	1543.10	1157.32	1928.87	Gamma	Local charge
RI	1797.48	1348.11	2246.84	Gamma	Local charge
LE	5069.89	3802.42	6337.36	Gamma	Local charge
Associated with drug administration					
Iintravenous infusion, per infusion	0.22	0.17	0.28	Gamma	Local charge
Antineoplastic drug allocation, per group	0.90	0.67	1.12	Gamma	Local charge
Cost of supportive drugs related to chemotherapy, per time^d^	2703.17	2027.38	3378.96	Gamma	Zhu et al., 2018 [29]
Average hospitalization cost related to chemotherapy (excluding drug fee), per time^d^	1454.95	1091.21	1818.69	Gamma	Zhu et al., 2018 [29]
Serious AEs (grade 3 and above), per unit					
Neutropenia, per unit^d^	815.10	0.00	815.10	Gamma	Chen et al., 2020 [24]
Thrombocytopenia, per unit^d^	605.63	0.00	605.63	Gamma	Chen et al., 2020 [24]
Febrile neutropenia, per unit^d^	4516.31	0.00	4516.31	Gamma	Chen et al., 2020 [24]
Hypertension, per unit	968.52	0.00	968.52	Gamma	The Writing Committee of the Report on Cardiovascular Health and Diseases in China 2022 [33]
Outpatient expenses of hypertension, per year	129.48	0.00	129.48	Gamma	Wang 2021 [34]
Routine follow-up of patients, per unit^e^	48.80	36.60	61.00	Gamma	The Guidelines for Diagnosis and Treatment of Chronic Lymphocytic Leukemia/Small Lymphocytic Lymphoma in China (2022) [7, 8]; Chen et al., 2020 [24]
BSC, per month	299.95	224.96	374.93	Gamma	Yu et al., 2021 [30]
Allo-HSCT	59621.17	44715.88	74526.46	Gamma	Zhang et al., 2021 [32]
End-of-life costs^d^	12455.19	11711.66	15682.52	Gamma	Zhu et al., 2018 [31]
Utilities					
Health state					
EFS, oral treatment	0.71	0.67	0.75	Beta	Kosmas et al., 2015 [36]
EFS, IV treatment	0.67	0.63	0.71	Beta	Kosmas et al., 2015 [36]
Progression after first-line therapy	0.66	0.62	0.71	Beta	Kosmas et al., 2015 [36]
Relapsed treatment lines	0.42	0.37	0.47	Beta	Kosmas et al., 2015 [36]
Death	0	-	-		
AEs					
Neutropenia	-0.163	-0.195	-0.12225	Beta	Tolley et al., 2013 [37]
Thrombocytopenia	-0.108	-0.135	-0.081	Beta	Tolley et al., 2013 [37]
Febrile neutropenia	-0.15	-0.1875	-0.1125	Beta	Chatterjee et al., 2021 [38]
Hypertension	-0.195	-0.195	-0.14625	Beta	NICE ID749 [39]
Risks for serious AEs (grade 3 and above), %					
Neutropenia in BE plus RI group	40.34	30.26	50.43	Beta	Woyach et al., 2018 [16]
Thrombocytopenia in BE plus RI group	14.77	11.08	18.46	Beta	Woyach et al., 2018 [16]
Febrile neutropenia in BE plus RI group	7.39	5.54	9.24	Beta	Woyach et al., 2018 [16]
Hypertension in BE plus RI group	14.20	10.65	17.75	Beta	Woyach et al., 2018 [16]
Neutropenia in IB group	15.00	11.25	18.75	Beta	Woyach et al., 2018 [16]
Thrombocytopenia in IB group	6.67	5.00	8.34	Beta	Woyach et al., 2018 [16]
Febrile neutropenia in IB group	1.67	1.25	2.09	Beta	Woyach et al., 2018 [16]
Hypertension in IB group	29.44	22.08	36.80	Beta	Woyach et al., 2018 [16]
Body surface area, m^2^	1.72	1.50	1.90	Normal	Chen et al., 2020 [24]
Discount rate	0.05	0	0.08	Fixed in PSA	Liu 2020 [15]

Abbreviations: IB, ibrutinib; ZB, zanubrutinib; OB, orelabrutinib; BE, bendamustine; RI, rituximab; LE, lenalidomide; CLL, chronic lymphocytic leukemia; PFS, progression-free survival; OS, overall survival; BSC, best supportive care; SD, standard deviation; ORR, objective response rate; HSCT, hematopoietic stem cell transplantation; EFS, event-free survival; IV, intravenous; Allo-HSCT, allogeneic hematopoietic stem cell transplantation; AEs, adverse events; PSA, probabilistic sensitivity analysis

^a^The range fitted low-high or 25%-range was performed for 1-way sensitivity analysis

^b^Parameters of Weibull models in different lines of strategies fitted to Kaplan-Meier survival curves derived from prior studies

^c^The costs of drug procurement (estimated in 2022 US dollars) came from the official maximum bidding prices published in the latest pricing negotiation of the Chinese National Health Insurance before November 8, 2023

^d^The base values of costs were adjusted to 2022 levels based on Chinese health component of Consumer Price Index

^e^According to the previous study and expert advices, patients were recommended to visit every 3 months in the first 2 years, every 6 months in the 3rd to 5th years, and then every year thereafter. The unit cost included physician-visit fees (3.7%), and laboratory testing expenses containing liver and renal function (5.5%), blood routine testing (30.8%) and cytological examination of bone marrow smear (60.0%)

The TF state was transient, so patients in this state would automatically move to the PF state after relapse or TF. Age-adjusted mortality observed in the general population from the Chinese Sixth Census was used to estimate transition probabilities between the PF state and the death state. Furthermore, the probability of entering the BSC state after progress was calculated from a previous study [23], and we also assumed that all patients who progressed after receiving allo-HSCT entered the BSC health state.

### Cost data

We only considered direct medical costs associated with medical management needed during treatment and routine follow-up, treatment of SAE (grade 3 and above) and end-of-life (EOL) costs for our chosen perspective. All unit costs were extracted from the published local literature or calculated based on local charges (Table 1).

The costs of drug procurement came from the official maximum bidding prices published in the latest pricing negotiation of the Chinese National Health Insurance before 8 November 2023 [28]. The medical costs of each treatment strategy were further assessed based on pivotal clinical trials [16, 18–22]. Supportive drug costs related to traditional treatments were the most influential factor in the economic burden of Chinese patients with CLL; therefore, we decided to include these expenses in the BE plus RI group [29]. Furthermore, patients with CLL required hospitalization during chemoimmunotherapy in the BE plus RI group due to intravenous drug administration over the first 6 cycles [28]. According to our previous study and expert advice, patients were recommended to visit every 3 months during the first 2 years, every 6 months in the 3rd to 5th years, and then every year thereafter [7, 24]. The unit cost of routine follow-up included physician visit fees and laboratory testing expenses [7, 24]. The costs of the health status of the BSC and the EOL care, as well as the unit cost of HSCT, were derived from a previous study from China [30–32]. Treatment and administration expenses were calculated based on Markov cycles and calendar time.

To estimate the costs of treatment-related toxicities, only SAE of grade 3 and above with a significantly different incidence between 2 groups was considered in the ALLIANCE (A041202) trial [16], consisting of neutropenia, thrombocytopenia, febrile neutropenia, and hypertension. We obtained the rates of these SAEs for each strategy and derived their unit costs from published studies or from the calculation of local charges presented in Table 1 [24, 33]. The unit costs of these SAEs were then multiplied by published rates to perform the model analysis. Furthermore, we assumed that SAE only occurred in the first month in the EFS state. Furthermore, for patients with CLL who experienced hypertension as a SAE, we estimated additional annual outpatient costs [34].

According to the Chinese health component of the Consumer Price Index (CPI) [35], all costs estimated before 2023 were adjusted to averages of 2022 and estimated in US dollars, assuming that the average exchange rate of 2022 was 1 US dollar to 6.7261 Chinese yuan.

### Utilities

We used utility scores to assess the burden related to patients with CLL. Utility scores reflected the value of the health-related quality of life (HRQoL) of a particular state of health. The HRQoL utility is typically summarized as a single score that presents a range of 1.00 (full health) to 0.00 (death) in the model. Due to the lack of published Chinese data on CLL-specific utilities, our utility scores were based on those of Kosmas et al. [36], a study that calculated the health-state utilities of the population of the United Kingdom specific to CLL by employing the time-trade-off (TTO) methodology. Based on this study, the EFS state provided the greatest utility during earlier lines of treatment in our model (Table 1). The PF state indicating progression after the first-line therapy or relapsed treatment lines was associated with a utility of 0.66 or 0.42, respectively. We also enrolled published disutilities associated with SAEs for each line of therapy [37, 38]. The disutility of hypertension entered the model as 0.195, which was the highest utility decrease assumed by the National Institute of Health and Care Excellence (NICE) since it was not available in published studies [39].

### Model outcomes

All costs and quality-adjusted life-years (QALYs) as model outputs were obtained after 10-year treatment. Incremental cost-effectiveness ratios (ICERs) were assessed by these model outputs, with a calculation method of dividing the total cost difference between the IB group and the BE plus RI group by the QALYs difference gained between these two groups, in terms of the incremental cost saved per QALY. If a more costly treatment scheme did not provide additional benefits compared with an alternative treatment scheme, then we believed that it was “dominated” by the alternative treatment scheme. If a more expensive treatment scheme provided additional benefits, then we compared the cost-effectiveness of the 2 arms by calculating an ICER.

### Sensitivity analysis

We performed a one-way sensitivity analysis and a probabilistic sensitivity analysis (PSA) to estimate the impact of parameter uncertainty on robustness in our model. All key parameters that fit low-high or 25% range values and specific distribution patterns in our model are presented in Table 1. Normal distributions were adopted for all input costs and BSA, and beta distributions were chosen for utilities and probabilities. Meanwhile, the discount rate was fixed in the PSA. The Chinese willingness-to-pay (WTP) threshold of 3 times the GDP per capita ($38,223.34) per QALY gained was used [15]. During the one-way sensitivity analyses, individual parameters were changed throughout their range to ascertain the impact on the ICER, and the results were expressed using tornado graphs. During the PSA, we conducted 1000 Monte Carlo simulations, randomly sampling from the distributions of model input at each time. The results of PSA were presented as a cost-effectiveness scatter plot and acceptability curves.

## Results

### Base-case analysis

First-line therapy with IB obtained an improvement of 1.17 QALYs compared to the BE plus RI strategy (4.48 vs. 3.30 QALYs, respectively). Furthermore, first-line BE plus RI treatment was associated with significantly higher health care costs ($272,088.31 vs. $184,041.53, respectively), with an incremental cost of $88,046.78 (Table 2). Therefore, the first-line IB strategy was absolutely dominant compared to the first-line therapy of BE plus RI in our cost-effective analysis.

**Table 2:** Base-case analysis of cost-effectiveness over 10 years for 2 groups

Variable	First-line of IB group	First-line of BE plus RI group	Incremental (vs. First-line of IB group)
No. of QALYs gained	4.48	3.30	-1.17
Costs, $^a^			
Costs of drugs			
Costs of first-line drugs	172,897.84	237,779.12	64,881.27
Costs of subsequent drugs	6,529.77	22,455.00	15,925.23
Costs of AEs	229.26	66.27	-163.00
Other Costs	4,384.66	11,787.93	7,403.27
Total costs, $	184,041.53	272,088.31	88,046.78
ICERs, $ per QALY	–	–	-75,107.35
Dominance	Absolute dominated	–	–

Abbreviations: IB, ibrutinib; BE, bendamustine; RI, rituximab; QALYs, quality-adjusted life-years; AEs, adverse events; ICERs, incremental cost-effectiveness ratios

^a^Costs are estimated in 2022 US dollars

### Sensitivity analysis

**Fig. 3:**
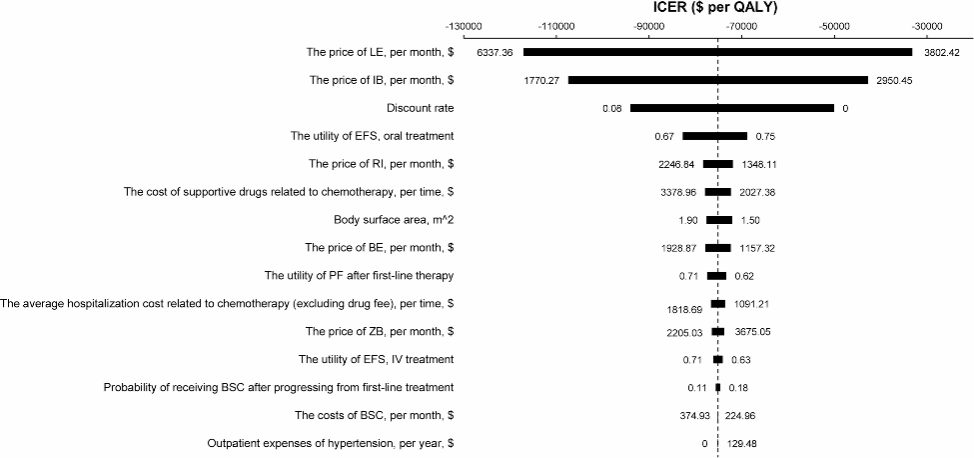
A one-way sensitivity analysis of incremental costs per quality adjusted life year (QALY) gained. Illustrated is the QALY for the first-line ibrutinib (IB) strategy versus the bendamustine (BE) plus rituximab (RI) strategy over a 10-year period (only the main 15 factors are listed). The bars represent the deviation in the basic case incremental cost-effectiveness ratios (ICERs) caused by various inputs of parameters into the model, as described in Table 1. LE: lenalidomide; ZB: zanubrutinib; EFS: event-free survival; PF: post-treatment failure; BSC: best supportive care; OB: orelabrutinib; QALY: quality adjusted life-year

During the one-way sensitivity analysis, either of the parameters in the inputs of the model had no substantial impact on the results. The 15 most sensitive variables associated with ICERs (the IB group versus BE plus RI group) are presented as a tornado graph in Fig. 3. All of the varying ICERs were negative below the Chinese WTP threshold of $38,223.34 per QALY gained. Among these variables, the price of LE per month, the price of IB per month, and the discount rate were considered the first three variables with the greatest impact on ICER.

**Fig. 4:**
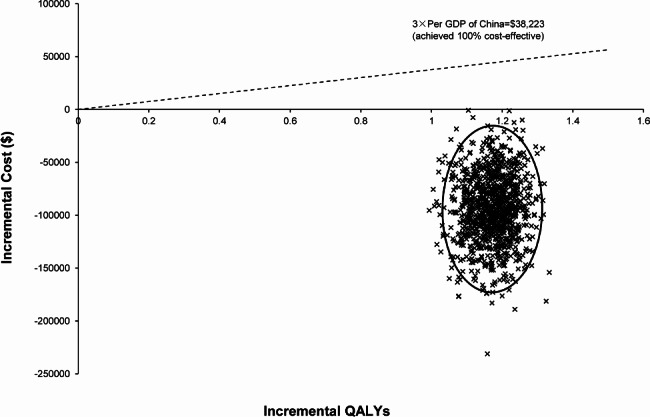
Cost-effectiveness plane of the first-line ibrutinib (IB) strategy versus the bendamustine (BE) plus rituximab (RI) strategy. The scatterplot of 1000 bootstrap replicas illustrates differences in the incremental cost-effectiveness ratios (ICERs) between the first-line IB strategy and the first-line BE plus RI strategy over a 10-year period. The diagonal dashed line indicates the Chinese willingness-to-pay (WTP) threshold of $38,223.34 per quality-adjusted life-year (QALY) gained. All simulations fell within the lower right quadrant of the cost-effectiveness plane: The IB regimen was less expensive and more effective than the BE plus RI regimen, containing 100% of the replicates. GDP: Gross domestic product

**Fig. 5:**
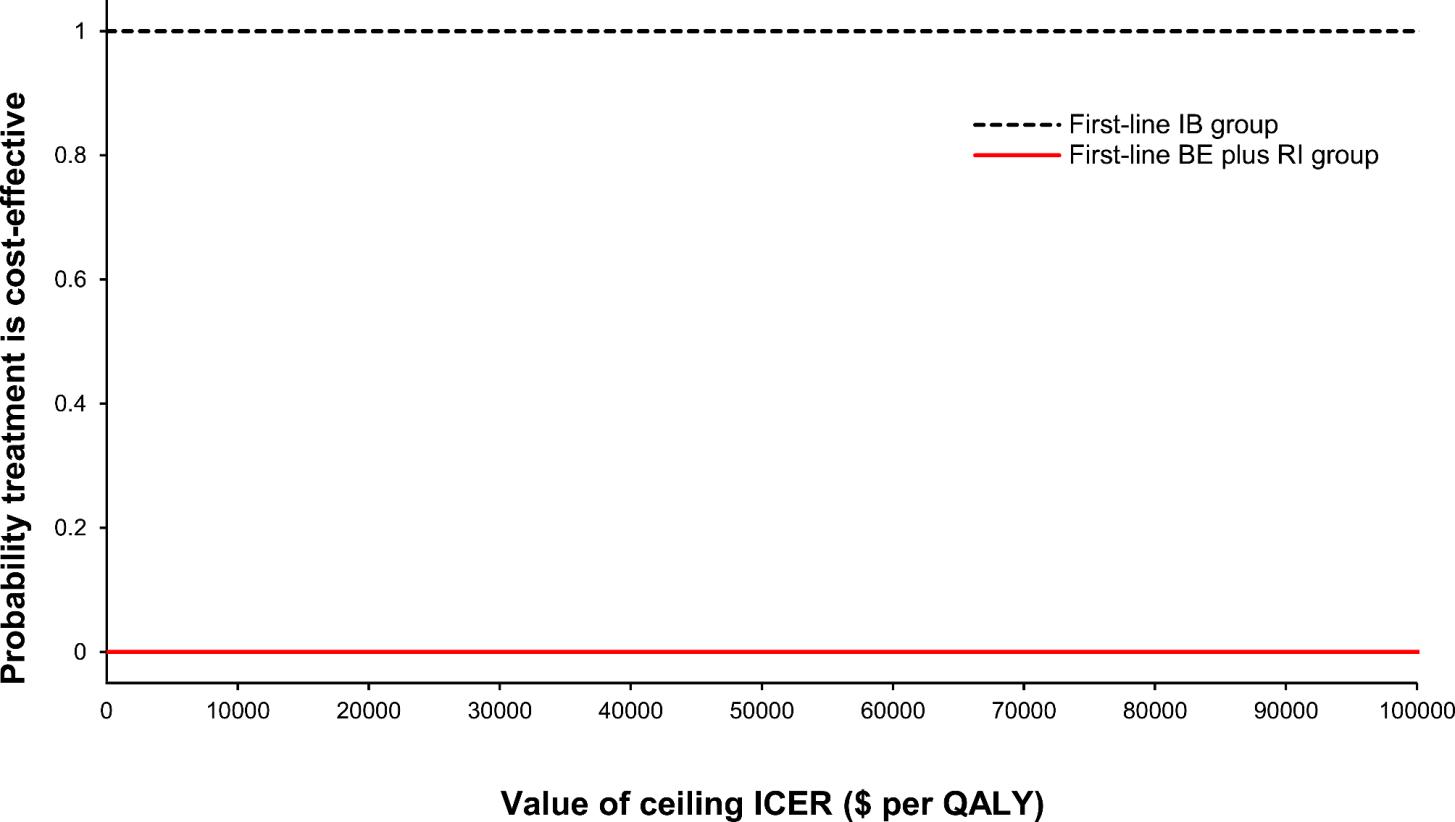
Cost-effectiveness acceptability curves based on 1000 iterations of the Markov model. Shown is the probability (y-axis) that the incremental cost-effectiveness ratios (ICERs) generated by the Monte Carlo simulations were lower than or equal to the ceiling ICER (x-axis). There was a 100% probability that the first-line ibrutinib (IB) strategy was cost effective at a Chinese willingness-to-pay threshold of $38,223.34 per quality adjusted life-year gained. BE: bendamustine; RI: rituximab; QALY: quality-adjusted life-year

The results of the PSA sampling from 1000 Monte Carlo simulations are described in Fig. 4, which confirms the robustness of our base-case results. In this scatterplot, 100% of the points were in the lower right quadrant, indicating that the IB regimen was less costly and more effective, that is, it presented absolute dominance. In Fig. 4, a diagonal dashed line indicates the Chinese WTP threshold ($38,223.34 per QALY gained). The acceptability curves illustrating cost effectiveness are shown in Fig. 5 and further confirmed that there was a 100% probability that the first line of the IB group showed cost effectiveness below the Chinese WTP threshold of $38,223.34 per gained QALY.

## Discussion

This was the first Chinese lifetime economic evaluation of the first-line IB strategy versus the BE plus RI strategy for the treatment of 65 or older patients with untreated CLL without the del (17p)/TP53 mutation from the perspective of Chinese health care system. Under the Chinese WTP threshold of $38,223.34 per QALY, the first-line IB strategy was absolutely cost-effective compared to the BE plus RI strategy, which was further confirmed by sensitivity analysis (Figs. 3 and 4). These findings provide the most recent local evidence for Chinese CLL guidelines and provide the latest recommendations for Chinese decision-makers.

Our research had important advantages. First, it was constructed on the basis of the results of a large randomized phase 3 trial, which directly compared IB and BE plus RI as the first-line treatments for patients with untreated CLL aged 65 years or older without the del (17p)/TP53 mutation. Second, for the second- or third-line treatment strategies, the clinical data used in the analysis were all derived from relapsed patients. Finally, we included the costs of support drugs related to chemotherapy, mainly costs relative to adjuvant chemotherapy and prevention of AEs in the model [29], so that it could better estimate the costs of patients receiving the chemoimmunotherapy program.

In recent years, a reform of the medical insurance system has been promoted in China. IB was approved in China in August 2017 and was introduced into the national medical insurance directory through a special negotiation on anticancer drugs in 2018, with a significant price reduction of more than 65%, and its contract was successfully renewed in 2020 [40]. As a result, the usual price per month of IB treatment was approximately $2,360, and it was much lower than the drug cost per month of BE plus RI regimen, which was close to $3,340 and then maintained around $5,069 (Table 1). On the other hand, the IB price in China was significantly lower than that reported in some studies of more than $10,000 per month [11–13]. To our knowledge, several other published studies conducted in other countries have also examined the cost-effectiveness of the IB versus the BE plus RI regimen as the first-line treatment of patients with untreated CLL [13, 41, 42]. Most of those studies used data from the ALLIANCE (A041202) trial [13, 41], which was the same study data that we used to directly compare the first-line IB strategy with the BE plus RI strategy. The ICER of first-line IB therapy derived from the study of United States was found to be $2,350,041 per QALY, which was not cost-effective at a WTP threshold of $150,000 per QALY gained, and it was further confirmed by sensitivity analyses [13]. In addition, its threshold analysis showed that if the cost of IB per month decreased by 72% to $3,535, first-line IB therapy would be cost-effective. Therefore, the absolute cost-effectiveness of the first-line IB strategy in our model was compelling, since the IB price per month is only approximately $2,360 in China at present. Furthermore, the first-line treatment of ibrutinib in the study of Iran was associated with 0.20 incremental QALYs and $251.48 cost-saving per patient and was therefore considered as the dominant strategy, whose conclusion was consistent with ours [41]. Next, we could further explore these treatment strategies for the healthcare system budget impact.

Several limitations of our present research must be considered. First, to our knowledge, there was only one randomized controlled trial (ALLIANCE (A041202)) reported the efficacy and safety of ibrutinib monotherapy versus bendamustine plus rituximab regimen in patients 65 or older years with untreated CLL patients without del(17p) [16]. And our hypothetical cohorts of patients were mirrored the cohort of individuals from that trial and derived data of efficacy and safety from that. It might cause a bias, although other economic studies had also adopted this method [13, 41]. Second, although most of our model included data from large, randomized trials, there was still uncertainty about long-term outcomes of novel drugs beyond the trial period. In our model, we used a fitted parametric survival model to extrapolate transition probabilities after the trial [13]. Third, the CLL treatment landscape is rapidly evolving. For instance, venetoclax-based therapy has emerged as a breakthrough treatment [43], which was absent from our model. However, there have been no direct comparison trials between IB and venetoclax as a first-line treatment for patients with untreated CLL. Furthermore, such an indication was unavailable in China and the latest guidelines had not recommended it as the preferred first-line therapy for CLL [7, 8]. Fourth, our cohort from a previous study included a small number of patients harboring chromosome 17p deletions (6.4%) [16]. However, there was no significant difference in the 17p deletion rate between the two arms, and also no direct study has included patients without the 17p deletion for the comparison of IB treatment outcomes with those of the BE plus RI strategy. Therefore, we believed that this small number would not affect our results as has also been considered by other studies [7, 13]. Fifth, because of a lack of data from China, utility estimates were obtained from a non-Chinese study from the UK [36], which had comprehensively reported utility values of various health states required for our research. The method for obtaining health utility values has been recognized in the latest pharmacoeconomic evaluation guidelines in China [15] and has also been widely applied in many studies [24, 44, 45]. In addition, we conducted a one-way sensitivity analysis and a PSA by using 25% range values, and moreover, our research results showed that none of them would affect the final results (Figs. 3 and 4, and 5). Finally, our model may have underestimated the toxicity of IB, as some real-world studies have reported that treatment withdrawal rates and adverse effects were significantly higher compared to clinical trial data [46, 47]. Furthermore, for patients with a history of hepatitis, treatment with IB may also lead to the reactivation of hepatitis virus infection [48, 49].

## Conclusions

From the perspective of Chinese payer, IB as a first-line treatment strategy is more cost-effective than the BE plus RI strategy for the treatment of older patients with previously untreated CLL not harboring the del(17p)/TP53 mutation. Therefore, we strongly suggest that Chinese health authorities adopt the former strategy for this patient subgroup with CLL.

## Data Availability

The datasets generated or analyzed during the current study are from the following published articles: (1) Woyach JA, Ruppert AS, Heerema NA, et al. Ibrutinib regimens versus chemoimmunotherapy in older patients with untreated CLL. N Engl J Med. 2018;379(26):2517–2528, doi: 10.1056/NEJMoa1812836., (2) Fink AM, Bahlo J, Robrecht S, et al. Lenalidomide maintenance after first-line therapy for high-risk chronic lymphocytic leukaemia (CLLM1): final results from a randomised, double-blind, phase 3 study. Lancet Haematol. 2017;4(10):e475-e486, doi: 10.1016/S2352-3026(17)30171-0., (3) Cull G, Burger JA, Opat S, et al. Zanubrutinib for treatment-naïve and relapsed/refractory chronic lymphocytic leukaemia: long-term follow-up of the phase I/II AU-003 study. Br J Haematol. 2022;196(5):1209–1218, doi: 10.1111/bjh.17994., (4) Xu W, Song Y, Wang T, et al. Updated results from the phase II study of orelabrutinib monotherapy in Chinese patients with relapsed or refractory chronic lymphocytic leukemia/ small cell leukemia [abstract]. Blood. 2020;136(Suppl. 1):26–27, 10.1182/blood-2020-134531, (5) Byrd JC, Brown JR, O’Brien S, et al. Ibrutinib versus ofatumumab in previously treated chronic lymphoid leukemia. N Engl J Med. 2014;371(3):213–223, doi: 10.1056/NEJMoa1400376., (6) Munir T, Brown JR, O’Brien S, et al. Final analysis from RESONATE: up to six years of follow-up on ibrutinib in patients with previously treated chronic lymphocytic leukemia or small lymphocytic lymphoma. Am J Hematol. 2019;94(12):1353–1363, doi: 10.1002/ajh.25638., and (7) Else M, Wade R, Oscier D, et al. The long-term outcome of patients in the LRF CLL4 trial: the effect of salvage treatment and biological markers in those surviving 10 years. Br J Haematol. 2016;172(2):228–237, doi: 10.1111/bjh.13824.

## Abbreviations

CLL: chronic lymphocytic leukemia
AAIR: age-adjusted incidence rate
BTK: Bruton’s tyrosine kinase
IB: ibrutinib
ZB: zanubrutinib
BE: bendamustine
RI: rituximab
GDP: gross domestic product
IGVH: immunoglobulin variable heavy chain
BSA: body surface area
OB: orelabrutinib
LE: lenalidomide
allo-HSCT: allogeneic hematopoietic stem cell transplantation
BSC: best supportive care
EFS: event-free survival
TF: treatment failure
PF: post-treatment failure
SAEs: severe adverse events
OS: overall survival
PFS: progression-free survival
KM: Kaplan-Meier
IPD: individual patient data
EOL: end-of-life
HRQoL: health-related quality of life
TTO: time-trade-off
QALYs: quality-adjusted life-years
ICERs: incremental cost-effectiveness ratios
PSA: probabilistic sensitivity analysis
WTP: willingness to-pay
